# Expression of XBP1s in peritoneal mesothelial cells is critical for inflammation-induced peritoneal fibrosis

**DOI:** 10.1038/s41598-019-55557-1

**Published:** 2019-12-13

**Authors:** An Liu, Qiong Song, Yong Zheng, Guoshuang Xu, Chen Huang, Shiren Sun, Lijie He, Lijuan Zhao, Meilan Zhou

**Affiliations:** 1 Outpatient Department, Xi’an Children’s Hospital, Xi’an, 710043 China; 2 Department of Nephrology, Shaanxi Second People’s Hospital, Xi’an, 710005 China; 3 Department of Nephrology, Xijing Hospital, The Fourth Military Medical University of People’s Liberation Army, Xi’an, 710032 China

**Keywords:** Endoplasmic reticulum, Peritoneal dialysis

## Abstract

Intraperitoneal inflammation is the most important determinant of peritoneal fibrosis in patients with long-term peritoneal dialysis (PD). Spliced x-box binding protein-1 (XBP1s), a major proximal effector of unfolded protein response (UPR) signaling, plays an indispensable role in inflammation. Our study demonstrated that the inflammatory factor interleukin-1β (IL-1β) dose- and time-dependently induced XBP1s upregulation and interleukin-6 (IL-6) secretion, as well as the expression of the fibrotic marker fibronectin. However, these effects were prevented by the IRE1 endonuclease inhibitor STF083010 since it time-dependently reduced IL-1β-induced Xbp1 mRNA splicing, XBP1s protein expression, inflammatory factor IL-6 secretion and the expression of the fibrotic marker fibronectin in human peritoneal mesothelial cells (HPMCs). The overexpression and knockdown of XBP1s in HPMCs had a similar effect on fibronectin expression. In a rat model of peritoneal inflammation, STF083010 significantly attenuated chlorhexidine digluconate-induced XBP1s and α-smooth muscle actin expression, as well as fibrotic tissue proliferation, in the peritoneum. Our results suggest that XBP1s is a strong pathogenic factor that mediates inflammation-induced peritoneal fibrosis in peritoneal dialysis.

## Introduction

Peritoneal dialysis (PD) is one of the major effective forms of renal replacement for the treatment of end stage renal diseases (ESRD)^1^, especially within the first five years of dialysis. The population of patients undergoing PD has recently begun to increase sharply throughout the world, particularly in developing countries such as China, due to the implementation of improved healthcare policies^2^. However, the advantages of PD gradually weaken after the first five years of dialysis because of chronic peritoneal dysfunction, which is characterized by inflammation, peritoneal fibrosis and neo-angiogenesis at the lesion site^3^.

Several factors contribute to peritoneal inflammation during long-term PD. These factors include (1) the inflammatory status of chronic kidney disease as a result of a reduction in the renal clearance of cytokines, fluid overload, immunological dysfunction and infectious comorbidity^4^, (2) long-term exposure of the peritoneal membrane to unphysiological dialytic solutions with high glucose and low pH^5^, (3) PD-related peritonitis^6^, (4) uremia-induced inflammation in the peritoneum^7^, and (5) protein energy wasting^8^. These factors are usually related to one other and eventually lead to peritoneal fibrosis. Therefore, intraperitoneal inflammation-induced peritoneal fibrosis is the greatest challenge for long-term PD therapy thus far.

Inositol-requiring enzyme-1α (IRE1α)/X-box-binding protein 1 (XBP1) signaling is the most important pathway for the unfolded protein response (UPR)/endoplasmic reticulum (ER) stress. It has been revealed that, upon ER stress, unconventional splicing of Xbp1 mRNA occurs to generate spliced Xbp1 (Xbp1s)^9^. Xbp1s encodes an active transcription factor, XBP1s, which drives the expression of a wide range of gene targets involved in the inflammatory process through both dependent and independent pathways of the UPR^9,10^. Thus, XBP1s is very likely to mediate multiple factors to induce intraperitoneal inflammation in long-term PD. In the present study, we found that the inflammatory factor interleukin-1β (IL-1β) induces XBP1s upregulation and IL-6 secretion, as well as the expression of the fibrotic marker fibronectin, whereas the IRE1 endonuclease inhibitor STF083010 blocks IL-1β-induced Xbp1 mRNA splicing and alleviates inflammation and fibrosis in human peritoneal mesothelial cells (HPMCs). The fibrotic factor fibronectin was also found to be regulated by XBP1s through the gain and loss of XBP1s in HPMCs. In a rat model of peritoneal inflammation, STF083010 significantly attenuated chlorhexidine digluconate (CG)-induced XBP1s expression, α-smooth muscle actin (α-SMA) expression and fibrotic tissue proliferation in the peritoneum. Our data strongly imply that XBP1s is a pathogenic factor that may mediate inflammation-induced peritoneal fibrosis in PD.

## Materials and Methods

### Ethics statement

The Ethics Committee for Animal Experiments of the Fourth Military Medical University (Xi’an, Shaanxi, China) approved all animal work, and the experimental protocols strictly complied with the National Institutes of Health guide for the care and use of Laboratory animals (NIH Publications No. 8023, revised 1978).

### Cell culture and treatment

The immortal human peritoneal mesothelial cell (HPMC) line (ATCC, Rockville, MD), which is an untransformed HPMC line with properties similar to those of primary cells, was cultured in Earle’s M199 medium with 10% fetal bovine serum (FBS, Gibco) at 37 °C in a 5% CO_2_ incubator as described previously^11–15^. Prior to all experiments, the cells were synchronized for 12 h in serum-free medium. Then, the cells were incubated in serum-free medium containing a normal level of glucose in the absence or presence of various concentrations of IL-1β (0.5–5 ng/ml; Pepro Tech, USA) for different incubation periods. A specific inhibitor of Xbp1 splicing, STF083010 (60 μmol/L; Sigma, USA), was added to the cells 30 min before IL-1β treatment^16^.

For XBP1s overexpression or XBP1 knockdown experiments, we used a lentiviral system according to our previous report^17^.

### Real-time PCR

Real-time PCR was performed as described previously^17^. Briefly, total RNA was extracted from human peritoneal mesothelial cells and reverse transcribed into cDNA using MMLV reverse transcriptase (Promega). Three separate cDNA samples were used, each one was measured in triplicate. Target-specific primers and Precision Master Mix with SYBR green (Primer Design) were added to each cDNA sample. The PCR reactions were run under the following cycling conditions: an initial denaturation step of 95 °C for 2 minutes followed by 40 cycles of 15 seconds denaturation (95 °C) and 35 seconds annealing/elongation at 60 °C. 18 S RNA was used as an internal control. The relative level of or fold change in target gene expression was normalized to the level of 18 S RNA and compared to a control sample. The PCR primers^10,17–19^ used in this study are listed in Table 1.

**Table 1:** PCR primers.

Gene	Sequence	Size (bp)
XBP1s		198
Sense	5′-GAGTCCGCAGCAGGTG-3′	
Antisense	5′-TCCTTCTGGGTA GACCTCTGGGAG-3′	
IL-6		171
Sense	5′-AGCCACTCACCTCTTCAGAACGAATTGACA-3′	
Antisense	5′-AGCATCCATCTTTTTCAGCCATCTTTG-3′	
IL-8		53
Sense	5′-AAACCACCGGAAGGAACCAT-3′	
Antisense	5′-GCCAGCTTGGAAGTCATGT-3′	
TNFα		116
Sense	5′-TCTGGGCAGGTCTACTTTGG-3′	
Antisense	5′-GGTTGAGGGTGTCTGAAGG-3′	
18S RNA		137
Sense	5′-CGCTTCCTTACCTGGTTGAT-3′	
Antisense	5′-GAGCGACCAAAGGAACCATA-3′	

### Western blot analysis and ELISA

The expression of XBP1s and fibronectin was assessed by western blot analysis using a modified protocol^17,20^. HPMCs were harvested at different time points and lysed in RIPA buffer (50 mol/L Tris-HCl, pH 7.6, 5 mol/L ethylenediaminetetraacetic acid, 150 mol/L NaCl, 0.5% Nonidet P-40, and 0.5% Triton X-100) containing a cocktail of protease inhibitors (leupeptin, aprotinin, antipain, and PMSF). Protein concentrations were measured with the Bradford assay. A total of 40 μg of total protein of each sample was separated by 8–10% SDS-PAGE and then transferred to a nitrocellulose membrane (Millipore, Glostrup, Denmark). The membrane was blocked overnight at 4 °C in blocking solution (1 M Tris-buffered saline with 5% non-fat milk and 0.02% Tween 20 (TBST)) and incubated for 4 h at room temperature with a primary antibody in TBST containing 1% bovine serum albumin. After three washes with TBST, the blots were incubated for 45 min at room temperature with HRP-conjugated IgG secondary Abs (1:2000; GE Healthcare Life Sciences) and then washed three times with TBST. Detection was performed using an ECL kit (Santa Cruz Biotech) according to the manufacturer’s instructions. The western blots shown are representative of at least three independent experiments. The primary antibodies used were as follows: anti-XBP1 (sc-7160, Santa Cruz, 1:1000), anti-fibronectin (F3648, Sigma, 1:500), and anti–β-actin (A5316, Sigma, 1:2000).

To measure IL-6 secretion by HPMCs, supernatants from the cell cultures were collected and analyzed with IL-6 ELISA kits (eBioscience) according to the kit instructions and well-established methods^20,21^.

### Animals

Twenty-five 8-week-old male Sprague-Dawley rats (200 to 230 g body weight) (The Fourth Military Medical University Animal Lab Center, Xi’an, China) were used in the present study; five were assigned to the control group, ten were assigned to the CG group, and ten were assigned to the CG and STF083010 treatment group. Peritoneal fibrosis was induced by the intraperitoneal injection of 0.1% CG in 15% ethanol dissolved in saline every other day for 3 weeks, as described with slight modifications^22,23^. STF083010 (1 mg/kg) was injected 2 h before CG injection. The control rats were injected with the same volume of saline. The rats were sacrificed after 3 weeks, and the peritoneal tissues were collected for western blot analysis, histological assessment and immunohistochemical analysis.

### Histological assessment and immunohistochemistry

The rat peritoneal tissues were fixed in 10% formalin and then embedded in paraffin. The 4-μm sections were stained with hematoxylin and eosin and Masson’s trichrome as previously described^22^.

Immunohistochemical analyses of XBP1s, F4/80, CD31 and α-SMA were performed by the same protocol used in the laboratory. Peritoneal tissue sections were washed twice with phosphate-buffered saline (PBS) and permeabilized with 0.1% Triton X-100 in PBS for 5 min at room temperature. After washing with PBS 3 times, the tissue sections were blocked for 20 min with 5% bovine serum albumin (BSA) in PBS. Primary antibody in 1% BSA in PBS was added overnight at 4 °C. Afterwards, the sections were washed twice in PBS and then incubated with secondary antibody in 1% BSA in PBS for 1 h at RT in the dark. The tissue sections were washed twice with PBS and mounted in Vectashield mounting medium. The sections were imaged on an upright fluorescence microscope (Leica).

### Statistical analysis

The data are presented as the mean ± standard deviation (SD). Analysis of variance (ANOVA) and unpaired t test were used to test statistical significance. The level of significance was set at P < 0.05.

## Results

### IL-1β-induced XBP1s expression in human peritoneal mesothelial cells

**Figure 1:**
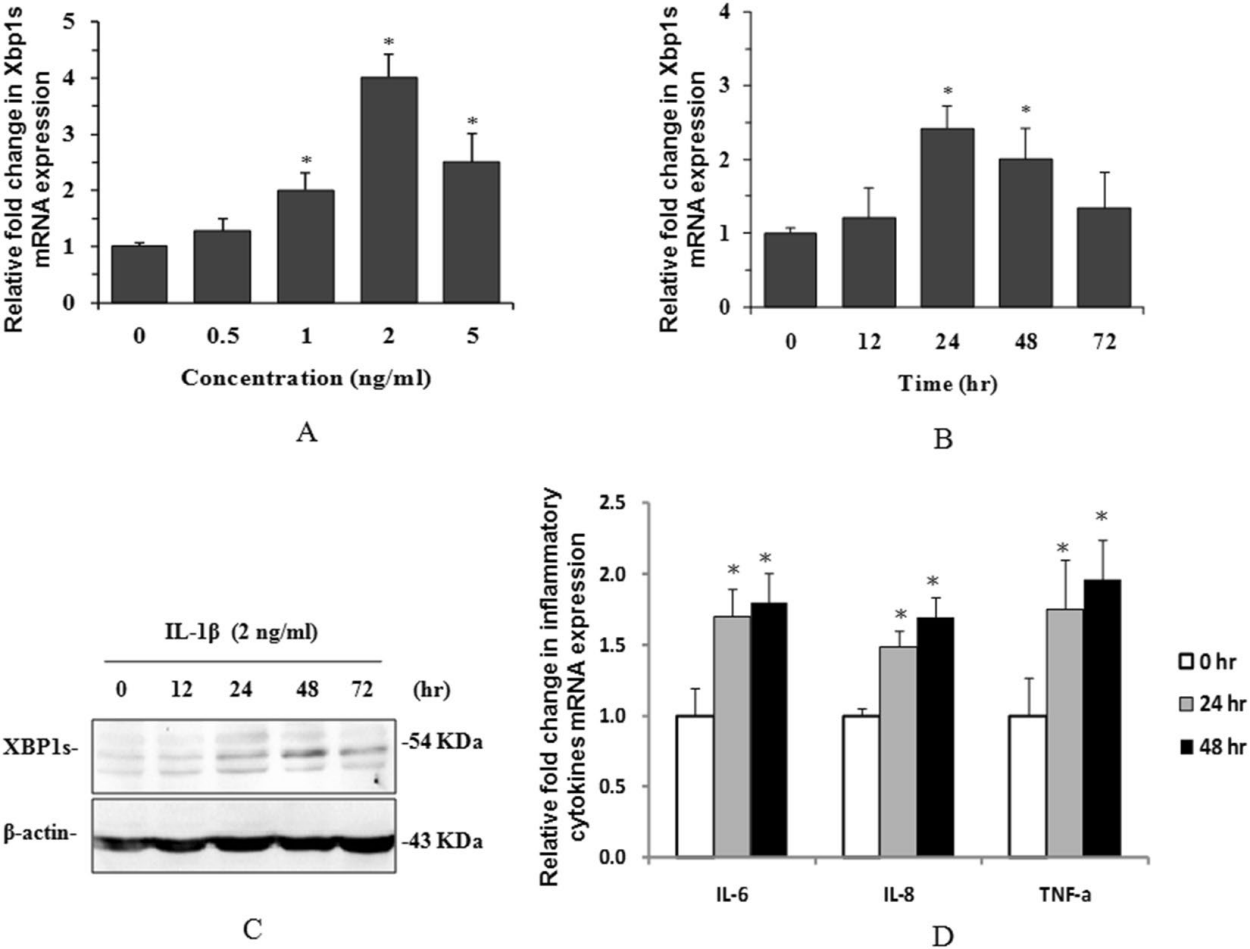
IL-1β induced XBP1s and inflammatory cytokine expression in HPMCs. Xbp1s mRNA was detected by real-time PCR after HPMCs were treated with IL-1β at different concentrations (**A**) and for different times (**B**). XBP1s protein expression was also detected by western blotting after HPMCs were treated with 2 ng/ml IL-1β for different times (**C**). Inflammatory cytokine mRNA expression was detected by real-time PCR after HPMCs were treated with 2 ng/ml IL-1β for different times (**D**). *P < 0.05 compared to the control group (n = 3 for each experiment). The full-length blots for (**C**) are shown in Supplementary Figure 1A.

To mimic inflammation in peritoneal dialysis, we treated human peritoneal mesothelial cells (HPMCs) with the inflammatory factor IL-1β to identify whether XBP1s is involved in this inflammatory process. The results indicated that IL-1β-induced Xbp1s mRNA expression in a concentration- (Fig. 1A) and time-(Fig. 1B) dependent manner. IL-1β also significantly induced XBP1s protein expression (Fig. 1C) in HPMCs. These results suggest that XBP1s is likely involved in the inflammatory process in HPMCs in peritoneal dialysis.

### XBP1s mediates IL-1β induced-inflammation and fibrosis in human peritoneal mesothelial cells

**Figure 2:**
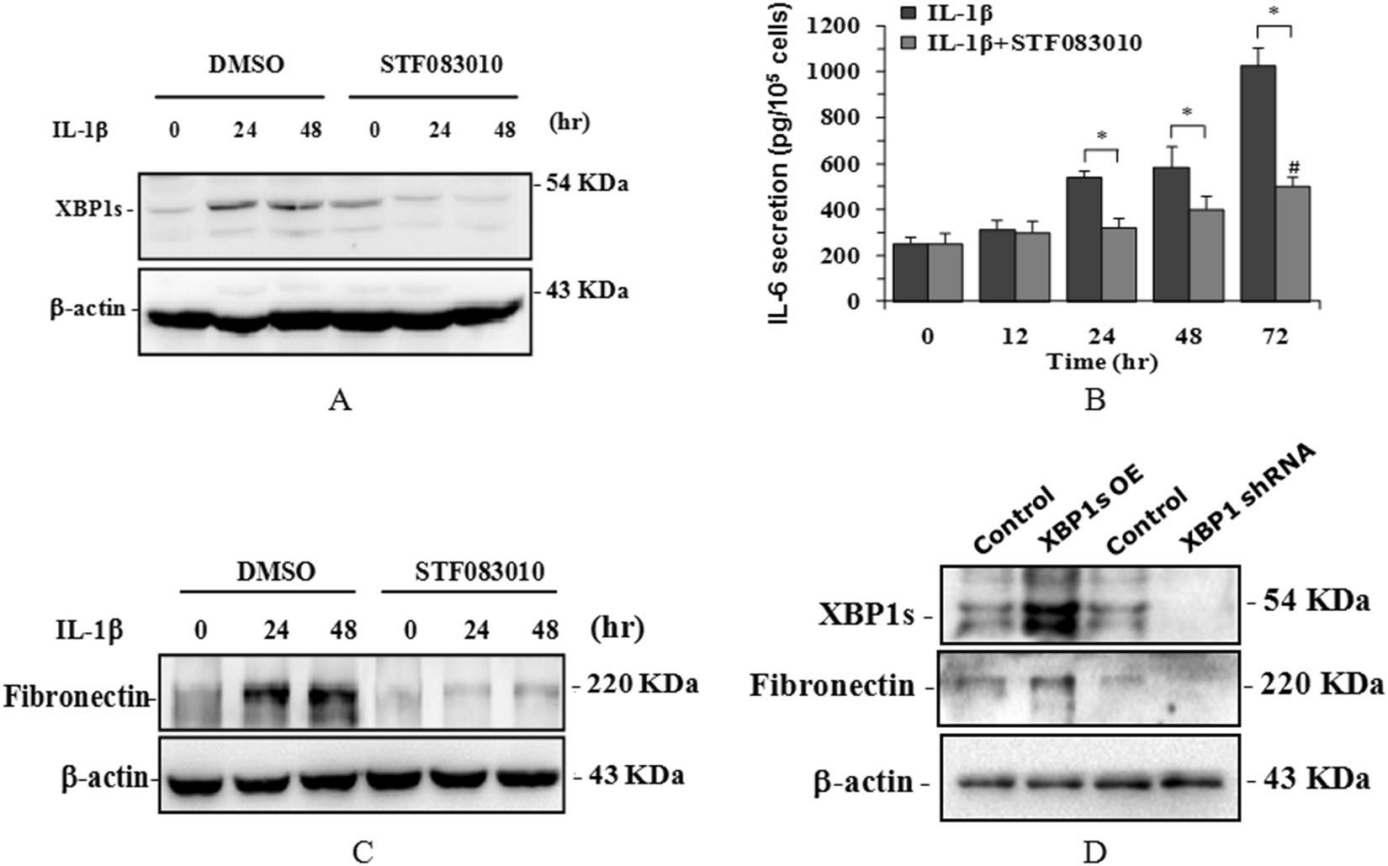
XBP1s-mediated inflammation and fibrosis in HPMCs. HPMCs were treated with 2 ng/ml IL-1β or pre-treated with STF083010 for different times. Then, XBP1s (**A**) and fibronectin (**C**) were detected in total cell lysates by western blotting, and IL-6 was detected in the culture supernatants by ELISA (**B**). The overexpression of XBP1s and the knockdown of XBP1 were achieved by the lentiviral infection of HPMCs, and then the XBP1s and fibronectin proteins were detected in total cell lysates by western blotting (**D**) *P < 0.05 compared to the control group (n = 3 for each experiment). The full-length blots for(**A**,**C**,**D**) are shown in Supplementary Fig. 1B–D.

To identify the function of XBP1s in IL-1β-induced inflammation and fibrosis, we first detected the gene expression of the inflammatory cytokines IL-6, IL-8 and TNFα. The results indicated that IL-1β strongly induced the expression of these cytokines (Fig. 1D). When the IRE1 endonuclease inhibitor STF083010 was used to inhibit Xbp1 splicing, IL-1β-induced XBP1s protein expression (Fig. 2A), as well as inflammatory factor IL-6 secretion (Fig. 2B) and fibrosis factor fibronectin expression (Fig. 2C), was significantly attenuated.

To further verify that STF083010 does inhibit IRE1 RNase activity and therefore inhibit XBP1 splicing to block inflammation-induced fibrosis in HPMCs, we directly interfered with XBP1s expression with gain and loss strategies using a lentiviral system. The results indicated that the overexpression of XBP1s strongly induced fibronectin expression, while the knockdown of XBP1 significantly decreased fibronectin expression (Fig. 2D).

These results demonstrate that XBP1s is critical in inflammation-induced fibrosis in human peritoneal mesothelial cells.

### XBP1s is involved in the inflammatory process in rat peritoneal tissue

**Figure 3:**
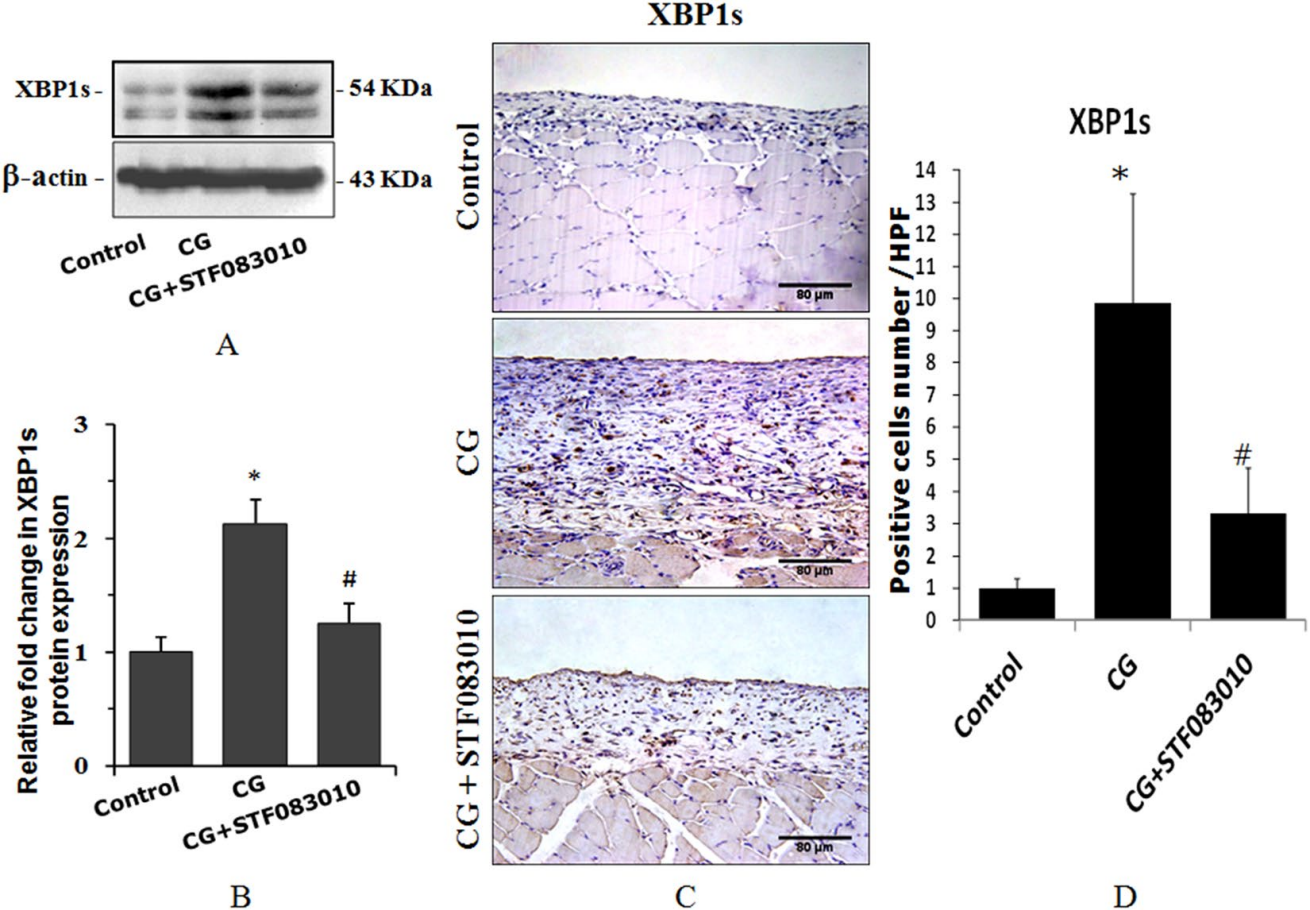
CG-induced XBP1s expression in rat peritoneal tissue. The rats were treated with intraperitoneal injections of 0.1% CG in 15% ethanol dissolved in saline or pretreated with intraperitoneal injections of STF083010 2 h before CG injection every other day for 3 weeks. Then, the XBP1s protein was detected by western blotting (**A,B**) and immunohistochemistry (**C,D**). The average number of pasitive cells in each high power field (HPF) was counted from 10 HPF of each group. *P < 0.05 compared to the control group. ^#^*P* < 0.05 compared to the CG-treated group (n = 5 for the control group, n = 10 for CG-treated group and CG + STF083010 group). The full-length blots for (**A**) are shown in Supplementary Fig. 2.

We injected the inflammatory reagent chlorhexidine gluconate (CG) into the rat abdominal cavity to mimic the inflammatory process in PD patients. The results of western blot analysis (Fig. 3A,B) and immunohistochemical staining (Fig. 3C,D) both indicated that XBP1s was strongly induced after 3 weeks of CG treatment, and this effect was dramatically inhibited by the intraperitoneal pre-injection of STF083010 2 h before the injection of CG, suggesting that XBP1s is involved in the inflammatory process of the rat peritoneum.

### XBP1s mediates chlorhexidine gluconate-induced inflammation and angiogenesis in rat peritoneal tissue

**Figure 4:**
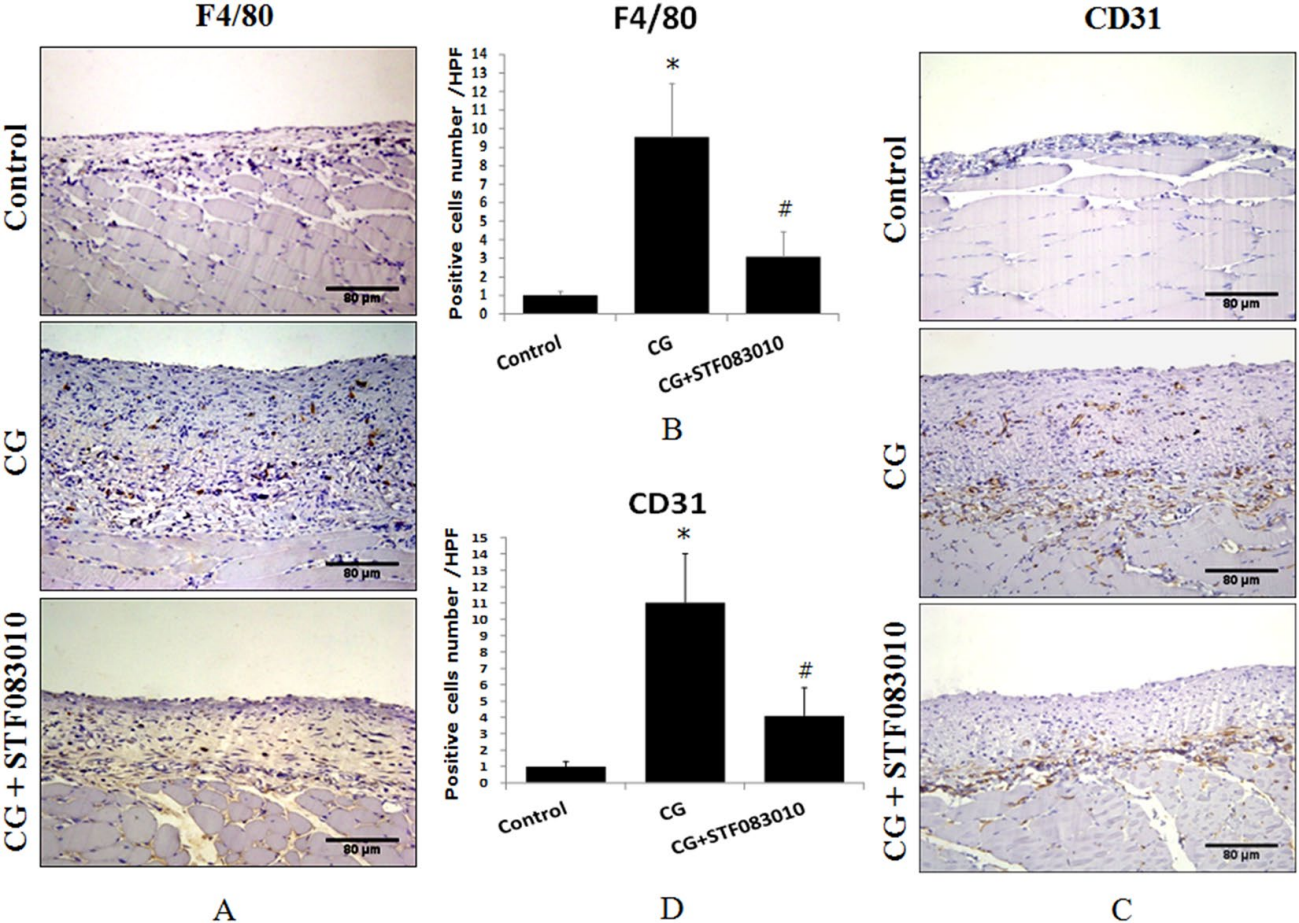
CG-induced F4/80 and CD31 expression in rat peritoneal tissue. The rats were treated with intraperitoneal injections of 0.1% CG in 15% ethanol dissolved in saline or pretreated with intraperitoneal injections of STF083010 2 h before CG injection every other day for 3 weeks, and then F4/80 (**A,B**) and CD31 (**C,D**) were detected by immunohistochemistry. The average number of pasitive cells in each high power field (HPF) was counted from 10 HPF of each group. *P < 0.05 compared to the control group. ^#^*P* < 0.05 compared to the CG-treated group (n = 5 for the control group, n = 10 for CG-treated group and CG + STF083010 group).

To identify the function of XBP1s in CG-induced inflammation and angiogenesis in the rat peritoneum, the macrophage marker F4/80 and platelet endothelial cell adhesion molecule-1 (PECAM-1/CD31) were detected by immunohistochemical staining of tissues from CG-treated rats and rats intraperitoneally pre-injected with STF083010. The results indicated that CG significantly induced F4/80 (Fig. 4A,B) and CD31 (Fig. 4C,D) expression, while the upregulation of these proteins was obviously attenuated by the pre-injection of STF083010, suggesting that XBP1s mediates CG-induced inflammation and angiogenesis in the rat peritoneum.

### XBP1s mediated chlorhexidine gluconate-induced fibrosis in rat peritoneal tissue

**Figure 5:**
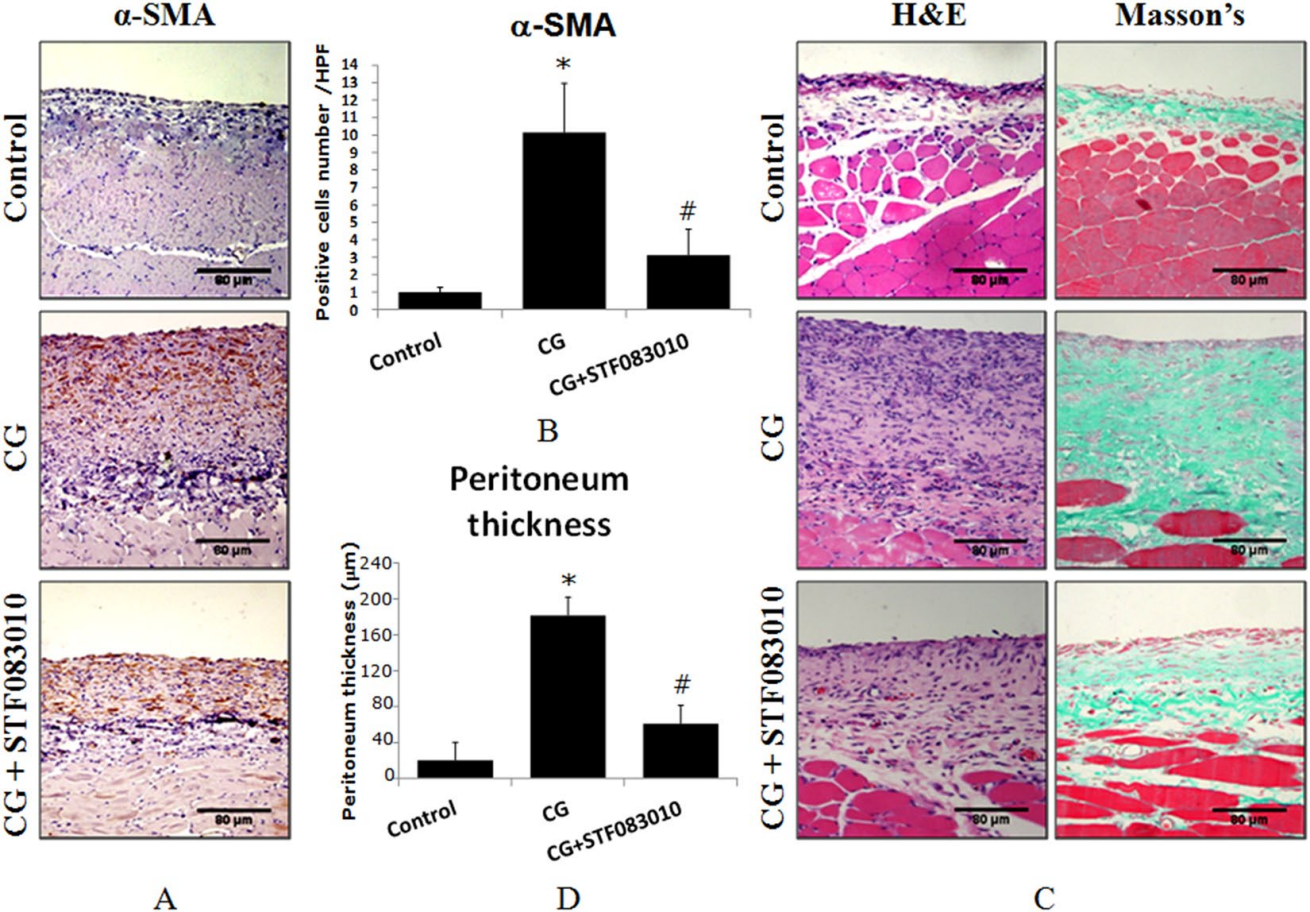
CG induced α-SMA expression and peritoneal tissue thickening. The rats were treated with intraperitoneal injections of 0.1% CG in 15% ethanol dissolved in saline or pretreated with intraperitoneal injections of STF083010 2 h before CG injection every other day for 3 weeks. Then, α-SMA was detected by immunohistochemistry (**A,B**). The average number of pasitive cells in each high power field (HPF) was counted from 10 HPF of each group. The peritoneal tissue thickness and peritoneal fibrosis were measured by H&E and Masson’s trichrome staining (**C,D**). *P < 0.05 compared to the control group. ^#^*P* < 0.05 compared to the CG-treated group (n = 5 for the control group, n = 10 for CG-treated group and CG + STF083010 group).

To verify the function of XBP1s in CG-induced rat peritoneal fibrosis, the fibroblast marker α-smooth muscle-actin (α-SMA) was first detected by immunohistochemical staining of tissues from CG-treated rats and rats intraperitoneally pre-injected with STF083010. Then, the peritoneal thickness and peritoneal fibrosis were also measured by H&E staining and Masson’s staining, respectively. The results indicated that CG significantly induced α-SMA expression (Fig. 5A,B), peritoneal thickening and peritoneal fibrosis (Fig. 5C,D), while these alterations were obviously attenuated by the pre-injection of STF083010. These results demonstrate that XBP1s is critical in inflammation-induced peritoneal fibrosis.

## Discussion

The greatest challenge for long-term peritoneal dialysis is inflammation-induced peritoneal fibrosis that results from the continuous exposure of the peritoneum to high glucose dialysate with low pH^24^. Therefore, a thorough understanding of the molecular changes of inflammation and fibrosis in the peritoneum is essential for reducing PD-related peritoneal fibrosis. In the present study, we showed that the UPR signaling molecule XBP1s was induced both at the mRNA (Fig. 1A,B) and protein (Fig. 1C) levels in human peritoneal mesothelial cells (HPMCs) upon treatment with the inflammatory factor IL-1β. The mRNA expression of the inflammatory cytokines IL-6, IL-8 and TNFα was also induced by IL-1β treatment (Fig. 1D), which is consistent with previous reports^25,26^. Moreover, IL-6 protein secretion and the expression of the fibrotic factor fibronectin were also synchronously induced by IL-1β. STF083010 is a specific blocker of IRE1α endonuclease activity, which inhibits IRE1α kinase activity therefore inhibits XBP1 splicing and without affecting IRE1α kinase function^27^. When exposed HPMCs to STF083010, the IL-1β-induced XPB1 splicing, as well as IL-6 secretion and fibronectin expression were greatly attenuated (Fig. 2A–C). These results suggest that IL-1β induced IRE1α-XBP1s pathway activation with activating IRE1α endonuclease activity and therefore induced XBP1 splicing. The XBP1s then promoted expression of inflammatory cytokines IL-6, IL-8 and TNFα, and finally facilitated expression of fibrotic factor fibronectin in HPMCs. Further XBP1 overexpression and knockdown experiments in HPMCs indicated that XBP1s positively controls fibronectin expression directly (Fig. 2D), suggesting that STF083010 attenuated fibronectin by inhibiting Xbp1 splicing. These observations clearly demonstrate that XBP1 signaling is involved in inflammation and fibrosis in HPMCs.

To identify the important role of XBP1s in PD-related peritoneal inflammation and fibrosis *in vivo*, we injected the inflammatory reagent chlorhexidine gluconate (CG) into the rat abdominal cavity to mimic the inflammatory process in PD patients. The results showed that CG dramatically induced XBP1s expression in rat peritoneal tissue, and this elevation was significantly suppressed by the Xbp1 splicing inhibitor STF083010 (Fig. 3A,B). Meanwhile, XBP1s also mediated CG-induced peritoneal fibrosis and the thickening of the peritoneal tissue (Fig. 5). These results demonstrate that XBP1s mediates inflammation-induced peritoneal fibrosis not only *in vitro* in HPMCs but also *in vivo*. It will be important to determine whether other cellular components of the peritoneal membrane, such as macrophages, also display enhanced XBP1s protein expression and whether XBP1s also plays a role in their involvement in peritoneal fibrosis in future studies.

To investigate the molecular mechanisms of the regulation of peritoneal fibrosis by XBP1s, we also detected another important inflammatory marker of macrophages, F4/80^28^, and the vascular endothelial cell marker CD31, which is a marker of angiogenesis in the process of peritoneal inflammation and fibrosis^22,23^, in peritoneal mesothelial cells. The results indicate that CG strongly induced F4/80 and CD31 expression in rat peritoneal tissue, while these effects were attenuated by the Xbp1 splicing inhibitor STF083010. Our data demonstrate that XBP1s is a pathogenic factor of peritoneal fibrosis that contributes to inflammation and angiogenesis in peritoneal dialysis.

As one of the most important pathways of the UPR, the IRE1α/XBP1 pathway plays a critical role in inflammation and cell survival^10,29^, and XBP1s controls inflammation through both UPR-dependent and UPR-independent pathways^10,30^. However, whether XBP1s also plays a critical role in peritoneal inflammation and fibrosis in peritoneal dialysis is still unclear. In the present study, we established a novel role for XBP1s in promoting peritoneal fibrosis by regulating inflammation and angiogenesis. Our results provide a clue for the search for therapeutic agents to potentially treat peritoneal dialysis-related peritoneal fibrosis by targeting XBP1s in future studies.

Nevertheless, we did not investigate the relationship between the XBP1s expression level in patient peritoneal tissues and peritoneal fibrosis. *In vivo* XBP1s loss- and gain-of-function studies are essential to further verify the critical role of XBP1s in peritoneal dialysis-related peritoneal fibrosis.

## Supplementary information

Supplementary Information
