# Supplementary material for: Expression of XBP1s in peritoneal mesothelial cells is critical for inflammation-induced peritoneal fibrosis
Source: Sci Rep. 2019 Dec 13;9:19043. doi: 10.1038/s41598-019-55557-1 (PMC6911080; doi:10.1038/s41598-019-55557-1)
Supplement: Supplementary file 1 — Supplementary Information [file 41598_2019_55557_MOESM1_ESM.pdf]

Expression of XBP1s in peritoneal mesothelial cells is critical for inflammation-induced peritoneal fibrosis

An Liu[a,1], Qiong Song[b,1], Yong Zheng[c,1], Guoshuang Xu[c*], Chen Huang[c], Shiren Sun[c], Lijie He[c], Lijuan Zhao[c], Meilan Zhou[c]

[a]Outpatient Department, Xi'an Children's Hospital, Xi'an 710043 China

[b]Department of Nephrology, Shaanxi Second People's Hospital, Xi'an 710005 China

[c]Department of Nephrology, Xijing Hospital, The Fourth Military Medical University of People's Liberation Army, Xi'an 710032 China

[1] These authors contributed equally to this work.

*Correspondence to:

Dr. Guoshuang Xu, Department of Nephrology, Xijing Hospital, The Fourth Military Medical University of People's Liberation Army, No.127, Changle West Road, Xi'an 710032, China. E-mail: xugsh882003@aliyun.com Telephone number: 86-18602916264

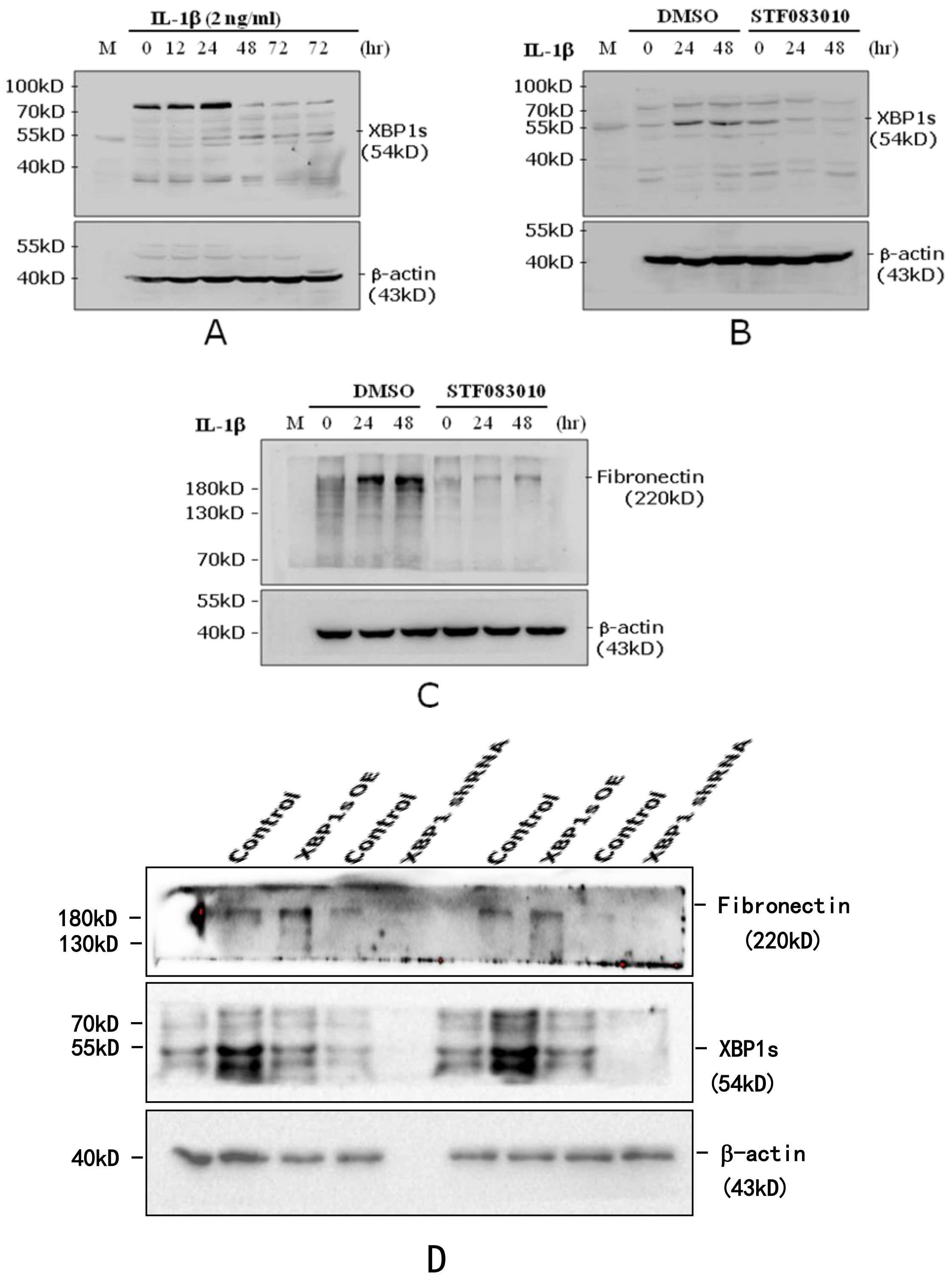

Supplementary Figure 1. IL-1β induced XBP1s and fibronetin expression (the full-length blots for Figure 1C, Figure 2A, 2C and 2D).

(A) The full-length blot for Figure 1C. The XBP1s was detected from the

full-length blot, while the β-actin detection was from a different part of the same blot (after detection of XBP1s, the blot was cropped between 70 kD and 55 kD, and β-actin was detected from the lower blot). (B) The full-length blots for Figure 2A. The XBP1s was detected from the full-length blot, while the β-actin detection was from a different part of the same blot (after detection of XBP1s, the blot was cropped between 70 kD and 55 kD, and β-actin was detected from the lower blot). (C) The full-length blot for Figure 2C. The fibronectin and β-actin were detected from different parts of the same blot (the blot was cropped between 70 kD and 55 kD, then finronectin was detected from the upper blot and β-actin was detected from the lower blot). (D) The full-length blot for Figure 2D. The fibronectin, XBP1s and β-actin were detected from different parts of the same blot (the blot was cropped into 3 pieces between 130 kD and 70 kD, 55 kD and 40 kD, then finronectin was detected from the upper blot, XBP1s was detected from the middle blot and β-actin was detected from the lower blot). M, protein marker. OE, overexpression. CG, chlorhexidine digluconate.

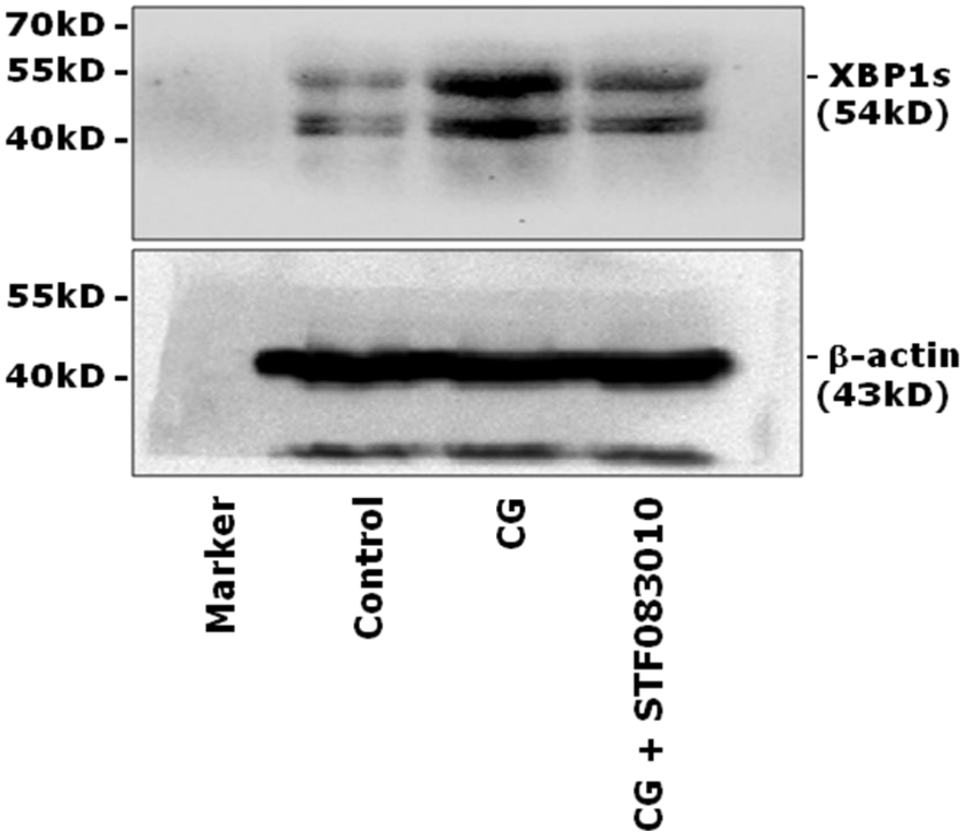

Supplementary Figure 2. CG induced XBP1s expression in rat peritoneal tissue (the full-length blots for Figur 3A). The XBP1s was detected from the full-length blot, while the β-actin detection was from a different part of the same blot (after detection of XBP1s, the blot was cropped between 70 kD and 55 kD, and β-actin was detected from the lower blot). CG, chlorhexidine digluconate.
